# Expectation shapes hunger and craving: placebo effects of verbal suggestion on food-related experiences

**DOI:** 10.1093/abm/kaag036

**Published:** 2026-06-19

**Authors:** Zaineb Al-Awan, Eleni Prinea, Aleksandrina Skvortsova

**Affiliations:** Health, Medical and Neuropsychology, Institute of Psychology, Leiden University, Wassenaarseweg 52, 2333 AK Leiden, The Netherlands; Department of Medical and Surgical Sciences, University of Bologna, Via Zamboni 33, 40126 Bologna, Italy; Health, Medical and Neuropsychology, Institute of Psychology, Leiden University, Wassenaarseweg 52, 2333 AK Leiden, The Netherlands; Health, Medical and Neuropsychology, Institute of Psychology, Leiden University, Wassenaarseweg 52, 2333 AK Leiden, The Netherlands

**Keywords:** placebo effect, hunger, food craving, verbal suggestion, appetite regulation, expectancy effects

## Abstract

**Background:**

Placebo effects—beneficial outcomes driven by expectations rather than active treatment—are well documented in domains such as pain, mental health, and fatigue. However, their impact on food metabolism and food-related behaviors remains underexplored.

**Purpose:**

This study investigated whether verbal suggestions about a sham vagus nerve stimulation could alter hunger, food craving, food desirability, and food choice in healthy volunteers.

**Methods:**

In a randomized, single-blind, between-subjects design (preregistered on Open Science Framework), 126 participants (101 women) were assigned to 1 of 3 groups: a hunger-increasing placebo group, a hunger-decreasing placebo group, or a control group. The hunger-decreasing group was told the stimulation would reduce hunger; the hunger-increasing group was told it would increase hunger; the control group received neutral information. Self-reported hunger and craving were assessed at 4 time points, alongside food desirability ratings and choices between natural and ultra-processed food options.

**Results:**

The hunger-decreasing group reported reduced overall craving compared to the control group and less hunger compared to the hunger-increasing group after food-related tasks. The hunger-increasing group showed increased hunger but no increase in overall craving. No effects were observed on food desirability or choice. The effect of the suggestions on hunger and craving was not mediated by expectations.

**Conclusions:**

These findings provide novel evidence that expectations shaped by verbal suggestion can influence food-related experiences, with potential implications for interventions targeting eating habits, eating disorders, and obesity prevention.

## Teaser

People’s expectations about a treatment can change their hunger and food craving, even when the treatment is not real. These findings could help support weight management and healthier eating habits.

The placebo effect refers to improvements in health, driven by positive expectations about a treatment. It has been shown to enhance the efficacy of interventions such as pain medication, antidepressants, and even surgery [1–3]. The nocebo effect, on the other hand, can be described as the negative counterpart of the placebo effect. It is defined by negative treatment outcomes that are caused, not by the active components of a treatment itself, but rather by negative expectations that an individual holds about the effects of the treatment [4].

Beyond these domains, emerging research suggests that placebo and nocebo effects may also influence hunger and food-related processes. For instance, a systematic review demonstrated that expectations about food can alter hormonal responses, with insulin and ghrelin levels changing in response not only to actual food, but also to related cues such as habitual meal times or food imagery [5]. Similarly, placebo responses induced through pharmacological conditioning of insulin were found to reduce hunger in normal-weight individuals [6].

Placebo effects have also been observed in clinical contexts. In a study on binge eating disorder, one-third of participants responded to placebo with fewer binge episodes, reduced symptom severity, and improved quality of life [7]. Experimental studies further support these effects: verbal suggestions about a placebo capsule influenced appetite, with satiety-enhancing suggestions reducing hunger, and hunger-increasing ones increasing ghrelin without affecting self-reported hunger [8]. Even open-label placebos—where participants are aware they are receiving an inactive treatment—have been shown to reduce appetite [9]. Additionally, placebo interventions have been found to reduce visual attention to food cues, as shown through eye-tracking [10] and selective attention tasks [11].

Despite growing evidence that placebo effects can influence hunger, it remains unclear whether they also affect other aspects of appetite, such as food cravings and choices between healthy or unhealthy food.

While hunger and craving are often used interchangeably, they serve 2 distinct functions. Hunger can be defined as a homeostatic need to eat and is regulated by the hypothalamus, whereas craving is an intense and specific desire for a particular food that is not necessarily related to hunger or nutritional needs [12, 13]. Craving is modulated by the brain’s reward circuitry and can be associated with addictive behaviors (drug craving, alcohol craving, food craving) [14]. Visual cues or mental imagery, hormonal fluctuations, and emotional states can induce feelings of craving even in the absence of hunger [15–17]. Importantly, craving is implicated in maladaptive eating behaviors, such as binge eating, which is marked by a feeling of loss of control during episodes of excessive food intake [18]. Therefore assessing craving as a distinct function alongside hunger can provide a more comprehensive understanding of eating behaviors and how they may be influenced by the placebo effects.

This study aimed to investigate the hunger-increasing and hunger-decreasing placebo effects induced by verbal suggestions on hunger and food cravings among healthy volunteers.

Given the fact that an increase in hunger is the opposite effect of the decrease in hunger we could refer to those as a nocebo and placebo effect, respectively. However, an increase in hunger is not necessarily a negative outcome in treatment and thus both conditions were treated as placebo effect in this study. It was hypothesized that (1) participants receiving hunger-decreasing suggestions, would experience less hunger and craving for their favorite food than the control group; and (2) participants receiving hunger-increasing suggestions, would experience more hunger and craving for their favorite food than the control group.

As secondary outcome measures, we hypothesized that (3) expectations would mediate the relationship between group and hunger and craving. We additionally hypothesized that (4) participants in the hunger-decreasing placebo group will give lower ratings for desirability of both high and low calorie items compared to participants in the control group; (5) participants in the hunger-increasing placebo group will give higher ratings for desirability of both high and low calorie items compared to participants in the control group. As previous research demonstrated that people might find unhealthy high calorie foods more desirable whilst hungry [19], we expected that (6) participants in the hunger-decreasing placebo group will choose less ultra-processed and more natural foods in the food choice task, compared to participants in the control group; and (7) participants in the hunger-increasing placebo group will choose more ultra-processed and less natural foods in the food choice task, compared to participants in the control group. Food desirability and food choice served as additional potential indicators for hunger and the real life eating behavior [20, 21].

## Methods

### Design

The study design and analyses were preregistered on Open Science Framework (https://osf.io/uj6f7). A randomized, single-blind, between-subjects design was used for this experiment. Participants were randomly allocated to 1 of 3 experimental groups: a hunger-decreasing placebo group, which received suggestions regarding the hunger-decreasing effects of a sham vagus nerve stimulation treatment; the hunger-increasing placebo group, which received suggestions about hunger-increasing effects of the sham treatment; and a control group, which received neutral suggestions. Participants in the experimental groups remained blinded regarding the group allocation, while experimenters were unblinded. Randomization was done in blocks separately for men and women, with the aim of having an equal number of men and women in each group.

### Participants

Data collection began on the 25th of March 2024 and concluded on the 21st of November 2024. Healthy volunteers between the ages of 18 and 35 years and of any gender were allowed to participate in the study. A cutoff age of 35 years was used because the sample consisted of university students, who are typically younger than this. This threshold helped prevent the inclusion of a small number of unusually older participants. The recruitment occurred primarily through the research management platform ‘Sona Systems’ that assigns credit scores for participation, as well as through flyers hung at university buildings. The convenience sampling method resulted in participants being university students, and no further information regarding their socioeconomic status was collected. A total of 126 participants (101 women and 25 men) were included in the study. The exclusion criteria of the study were the following: a body mass index less than 18 or greater than 29.9 (as measured in the lab), an official diagnosis of diabetes type I or II, a history or current diagnosis of an eating disorder, being pregnant, having eaten less than 2 h before the session, having drank alcohol or used recreational drugs in the last 12 h before the session. The effect size (η^2^ = 0.09), drawn from an earlier study reporting an interaction of similar design [22], was used to calculate the sample size. Power was estimated for the Group × Time interaction with 4 measurement moments using G*Power’s ‘repeated‑measures, within–between interaction’ test, based on the formula of Cohen [23]. It indicated that 126 participants (42 per group) were needed for 80% power at α = .05.

### Procedure

The study procedure is shown in Electronic Supplementary Material 1. Participants were told the study investigated the effects of a vagus nerve stimulator on food perception and hunger. After signing up, they attended a 1-h laboratory session and were instructed to fast for at least 2 h and abstain from alcohol and recreational drugs for 12 h prior. Upon arrival, participants provided informed consent, had their height and weight measured, and were screened for inclusion. They also named their favorite meal, used later in the Food Imagery Task and craving assessments. Next, participants completed the first set of questionnaires. The experimenter then introduced the vagus nerve stimulator and delivered verbal suggestions aligned with group allocation: hunger-decreasing, hunger-increasing, or control. Control participants were told they would receive a sham stimulation. All participants received a 2-min sham stimulation behind the ear, using the “Cerbomed Nemos” TENS device. Stimulation intensity was adjusted individually (maximum 0.7 mA) to be perceivable but not uncomfortable. Following this, participants completed the next set of questionnaires. They then performed a brief Food Imagery Task involving their favorite food, followed by a repeated hunger and craving questionnaire. Finally, participants completed the Food Desirability Task and Food Choice Task, and filled out the hunger and craving questionnaire once more. On the last page of the questionnaire, the participants were asked what they believed the study was about. None of them suspected that the study was about the placebo effect. At the end, they were debriefed by the researcher, received their preferred snack, and were compensated with €8.50 or 2 study credits. The session took approximately 1 h.

### Verbal suggestions

Participants in the (1) hunger-decreasing placebo group were told the study investigated the effects of vagus nerve stimulation on hunger, that previous research demonstrated that vagus nerve stimulation decreases hunger and that their hunger was expected to decrease after vagus nerve stimulation; (2) In the hunger-increasing placebo group, participants were told that previous research demonstrated that vagus nerve stimulation increases hunger and that their hunger was expected to increase after vagus nerve stimulation; (3) In the control group, participants were informed about the fact that they are in the control group and that no real vagus nerve stimulation will take place. The full text of the suggestions is presented in Electronic Supplementary Material 2.

### Measures

#### Hunger and food craving

Hunger was measured with a Visual Analogue Scale and a question that asked the participants how hungry they were. The scale had 2 anchors: “Not hungry at all” and “Extremely hungry”. The answers were coded from 0 to 100 but no numeric values were displayed to participants.

Food craving was measured similarly; the question asked how much they want to eat their favorite food right now, with a Visual Analogue Scale underneath ranging from 0 (“Not at all”) to 100 (“Extremely”). The 2 variables are measured at baseline, after the vagus nerve stimulator application (T1), after the food imagery task (T2), and lastly, after the computer tasks at the end of the experiment (T3).

#### Expectations

Participants were asked to rate how much they expected the vagus nerve stimulation to affect their hunger and food craving. This was measured with a Visual Analogue Scale with anchors “It will increase my hunger to a great extent” (on the left), “It will have no effect on my hunger” (in the middle) and “It will decrease my hunger to a great extent” (on the right). The answers were coded from −50 to 50 but no numeric values were displayed to participants. A score of 0 was captioned with the assumption that the stimulation would have no effect on hunger/craving, while lower scores indicated expectations of decreased hunger/craving and higher scores indicated expectations of increased hunger/craving.

#### Food imagery tasks

Participants were asked to follow instructions presented on a computer screen. The slides described a detailed scenario that asked participants to vividly imagine their favorite food, its structure, smell, and taste, for 3 min. An image of their favorite food, which was retrieved from Google Images, was included in the last slide. This was in order to induce short-term food cravings. Similar tasks have been previously demonstrated to induce food craving [15, 24].

#### Food desirability task

Participants were presented with 40 images of either ultra-processed or minimally processed foods in random order. The photos were taken from a standardized database [25] pretested in the Netherlands for recognizability and liking. After each image, participants rated their desire to eat the food on a VAS scale from “not at all” to “extremely,” with scores ranging from 0 to 100, where higher scores indicated greater desirability. In this context, natural or minimally processed foods (eg, fruits, vegetables, nuts, and cheese) were defined as ‘healthy’ foods, while ultra-processed foods (eg, candy, chips, and cookies) were defined as ‘unhealthy’ foods. Two mean scores were calculated for each participant: the average rating for the healthy foods and the average rating for unhealthy foods.

#### Food choice task

A validated food choice task [25] was used to measure the participants’ choice between ultra-processed unhealthy foods and natural healthy foods. Participants were presented with 61 pairs of natural and ultra-processed food pictures from the Natural & Ultra Processed Foods database [25]. They were asked to choose which of the items in each of the pairs they would prefer to eat right now. They were informed beforehand that they would receive one of the chosen items after the experiment. The item that was given to them was either an orange or a chocolate, depending on the choice they made in the task. The main outcome variable was the proportion of healthy choices, calculated as the number of healthy items chosen divided by the total amount of choices made.

#### Questionnaires

Body image was assessed with the Body Shape Questionnaire [26]. The total score of the 27 items ranged from 27 to 135, with higher numbers indicating a more negative body image. The questionnaire achieved a high test–retest reliability coefficient of .88, *P* < .001 [27].

Mood was measured using a shortened version of the Positive and Negative Affect Schedule (PANAS) [28]. The questionnaire consisted of 5 items measuring positive affect and 5 items measuring negative affect, with an internal consistency reliability score ranging from .84 to .90, *P* < .001 [29].

Impulsivity was assessed with the Barratt Impulsiveness Scale-15 (BIS-15) [30]. The scale consists of 15 self-rating items measuring components of impulsivity, and reached a test–retest reliability of .79, *P* < .001 [31].

Personality traits were measured using the Big Five Inventory, covering Extraversion, Agreeableness, Conscientiousness, Neuroticism, and Openness [32]. The internal consistency reliability scores of the subscales ranged from .73 to .80 [33].

Happiness was measured as a trait with the Oxford Happiness Questionnaire, with a reported internal consistency reliability score of .90 [34].

### Statistical analysis

The statistical analysis was performed in R. The analyses were preregistered, and the deviations of the pre-registration are noted in the following sections.

Assumptions of normality (Shapiro–Wilk test) and homogeneity of variance (Levene test) were evaluated. When homogeneity was violated, Welch ANOVA was used. Significant group effects were followed by Tukey tests for parametric data and Dunn with Bonferroni correction for non‑parametric data.

Group differences in baseline characteristics (BMI, age, hunger, craving, affect, body image, personality, and happiness) were assessed using 1‑way ANOVAs or Kruskal–Wallis tests, depending on normality.

To look at the effect of the suggestions on hunger over time, a repeated measures ANOVA with 4 measurement moments as a within-subjects factor and group as a within-subjects factor was used. We have also added a nonpreregistered analysis in which a hunger change score (mean of T1–T3 minus baseline) was compared between the groups with a one-way ANOVA. This was done to facilitate the interpretation of overall change. The same analyses were performed for craving.

Kruskal–Wallis test was done to compare 3 groups in hunger expectations, whereas a one-way ANOVA was used to compare the groups in craving expectations.

To investigate whether expectations mediated the effects of the suggestions on hunger change score, a mediation analysis was run using the regression-based approach of Hayes & Preacher (PROCESS Model 5) [35]. The independent variable was entered as a multicategorical predictor using indicator coding, with the Control condition serving as the reference group. This approach simultaneously estimates the relative indirect effects of the hunger-decreasing and hunger-increasing conditions compared to the Control group within a single model. Indirect effects were estimated using 5.000 bias-corrected bootstrap resamples to obtain 95% confidence intervals. The same mediation analyses were performed for the craving change score.

Two one‑way ANOVAs tested group differences in desirability ratings for healthy and unhealthy foods. Another one‑way ANOVA compared the proportion of healthy foods chosen across groups. Associations between T2 hunger and craving, desirability of the food items, and the proportion of healthy foods chosen in the Food Choice Task were examined using Spearman’s rank-order correlations.

An exploratory moderation analysis was performed as part of the preregistered analytical plan, the results can be found in the Supplementary Material 4.

## Results

### Baseline characteristics 1

Table 1 contains all means (*M*) and standard deviations (*SD*) of the measured baseline variables. No baseline difference among the 3 experimental groups was found on any of the characteristics, except age.

**Table 1:** Mean and standard deviation of baseline variables.

Variable	Overall		Hunger-decreasing Group		Control Group		Hunger- increasing Group		F(df)/χ^2^(df)*	*P*	η²
	M	(SD)	M	(SD)	M	(SD)	M	(SD)			
**Age**	21.3	(3.4)	22.2	(3.3)	20.7	(3.2)	20.8	(3.6)	8.87(2)*	.012	.071
**BMI**	22.4	(2.5)	22.6	(2.8)	22.2	(2.3)	22.4	(2.4)	0.35(2)*	.841	.003
**Hunger baseline**	48.2	(21.4)	48.7	(20.3)	49.9	(22.4)	45.5	(21.7)	0.45(2)	.639	.001
**Craving baseline**	66.2	(22.3)	70.5	(23.4)	65.7	(21.9)	61.8	(21.1)	5.22(2)*	.074	.042
**Positive Affect**	15.5	(3.7)	15.7	(3.9)	15.0	(3.6)	15.9	(3.4)	0.75(2)	.477	.010
**Negative Affect**	7.2	(2.6)	7.2	(2.4)	7.1	(2.7)	7.2	(2.8)	0.05(2)*	.976	.001
**Happiness**	122.1	(18.5)	125.0	(18.4)	118.4	(21.3)	122.9	(14.5)	2.59(2)*	.273	.021
**Body Image**	108.4	(16.1)	108.8	(14.4)	106.7	(17.1)	109.9	(17.1)	0.40(2)	.669	.001
**Impulsiveness**	30.9	(5.9)	31.6	(6.4)	30.8	(6.3)	30.3	(4.8)	0.47(2)	.626	.001
**Extraversion**	27.0	(6.3)	28.2	(5.3)	26.1	(7.9)	26.6	(5.0)	1.47(2)	.235	.035
**Agreeableness**	35.1	(5.3)	35.2	(5.1)	34.8	(5.8)	35.3	(4.9)	0.12(2)	.887	.001
**Conscientiousness**	30.8	(6.1)	31.1	(6.1)	30.2	(6.4)	31.3	(6.0)	0.88(2)*	.643	.007
**Neuroticism**	25.4	(6.3)	25.3	(6.9)	25.0	(6.4)	25.9	(5.6)	0.23(2)	.796	.001
**Openness**	36.8	(5.2)	36.9	(5.4)	36.5	(5.0)	36.9	(5.4)	0.08(2)	.923	.001

M = mean; SD = standard deviation.

Possible range of variables: Age = 18–35, BMI = 18–29.9, Baseline Hunger and Craving = 0–100, Positive and Negative affect = 5–25, Happiness = 29–174, Body Image = 27–135, Impulsiveness = 15–60, Extraversion = 8–40, Agreeableness = 9–45, Conscientiousness = 9–45, Neuroticism = 8–40, Openness = 10–50.

### Hunger

Table 2 showcases the means and standard deviations of hunger and food craving scores post-manipulation.

**Table 2:** Mean and standard deviation of hunger and food craving post-manipulation.

Timepoint	Variable	Overall		Hunger-decreasing Group		Control Group		Hunger-increasing Group	
		M	(SD)	M	(SD)	M	(SD)	M	(SD)
**1**	Hunger	48.25	(22.93)	43.18	(23.42)	48.66	(22.19)	53.66	(22.49)
	Craving	56.35	(25.78)	51.24	(27.25)	57.45	(25.65)	60.97	(23.72)
**2**	Hunger	59.25	(23.78)	52.50	(24.36)	61.02	(21.66)	65.00	(24.14)
	Craving	71.21	(23.15)	64.86	(26.12)	74.41	(19.67)	74.87	(22.23)
**3**	Hunger	62.45	(23.06)	54.34	(22.31)	62.91	(22.33)	71.32	(21.91)
	Craving	67.92	(23.65)	60.82	(25.79)	71.11	(21.12)	72.45	(22.46)

**Figure 1:**
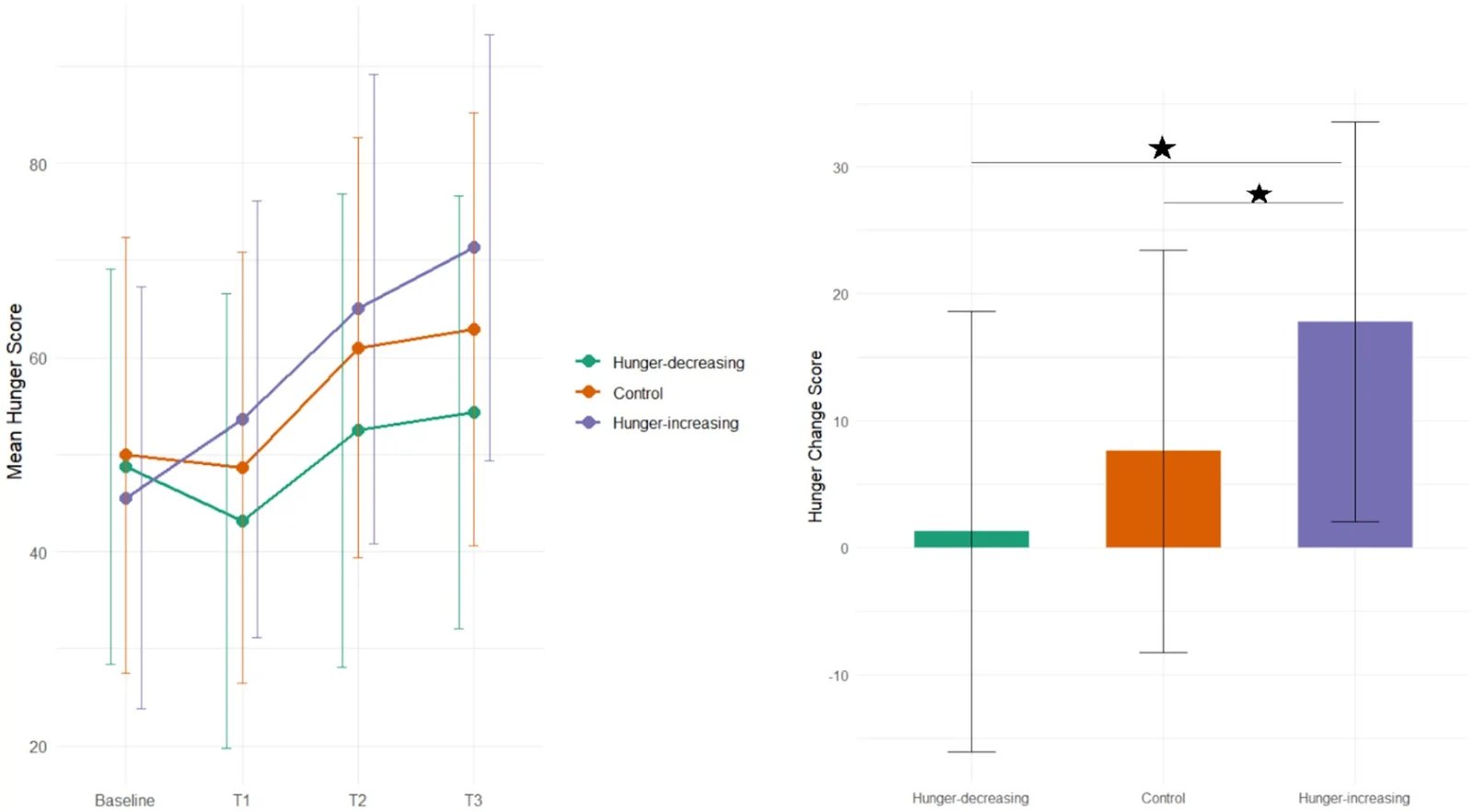
Means and SDs of hunger scores per group over time (left) and mean hunger change score per group (right).

A significant group effect was found on hunger change scores, *F*(2, 123) = 10.51, *P* < .001, eta squared (η^2^) = 0.15. Post-hoc comparisons (family-wise error corrected) showed that the hunger-increasing group reported significantly more hunger than both the control (mean difference = 10.20, 95% CI [1.61, 18.79], *p*-adjusted = .015) and the hunger-decreasing group (mean difference = 16.52, 95% CI [7.93, 25.11], *p*-adjusted < .001). The hunger-decreasing group did not significantly differ from the control (mean difference = –6.32, 95% CI [–14.59, 1.95], *p*-adjusted = .170). Mean scores are shown in Figure 1 (right).

A group × time interaction was also significant, *F*(6, 366) = 6.02, *P* < .001, η^2^*g *= 0.020, indicating that hunger changed differently across groups over time (Figure 1, left). One-way ANOVAs per timepoint revealed no significant group differences at baseline, T1, or T2 (*p*-adjusted > .098), but a significant effect emerged at T3, *F*(2, 122) = 5.81, *p*-adjusted = .016, η^2^*g *= 0.087. Pairwise comparisons showed the hunger-increasing group had significantly higher hunger than the hunger-decreasing group (*p*-adjusted = .002).

There was also a significant main effect of time, *F*(3, 366) = 52.30, *P* < .001, η^2^*g *= 0.081, indicating hunger increased over time. The main effect of the group was not significant, *F*(2, 122) = 2.19, *P* = .116, η^2^*g *= 0.028.

The outcomes of the analysis were unchanged when age was added as a covariate (see Supplementary Material 4).

### Food craving

**Figure 2:**
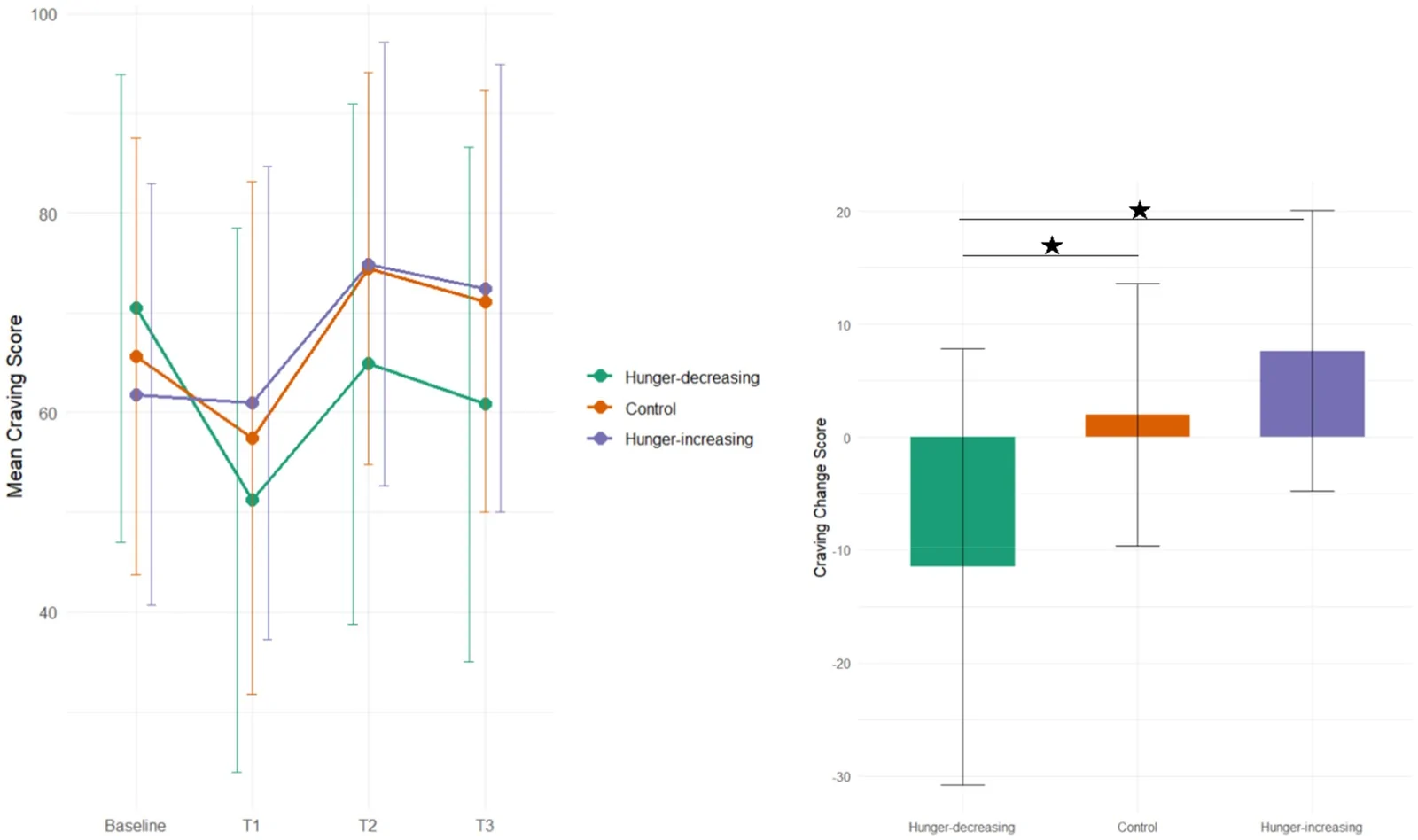
Means and SD craving scores per group over time (left) and the mean craving change score per group (right).

Group differences in craving change scores were significant, *F*(2, 123) = 17.98, *P* < .001, η^2^ = 0.23. Post-hoc tests showed the hunger-decreasing group had significantly lower craving than both the control (mean difference = –13.48, 95% CI [–21.05, –5.90], *p*-adjusted < .001) and hunger-increasing group (mean difference = 19.12, 95% CI [11.25, 26.99], *p*-adjusted < .001). The hunger-increasing and control groups did not significantly differ (mean difference = 5.64, 95% CI [–2.23, 13.51], *p*-adjusted = .210). See Figure 2 (right) for mean scores.

A significant group × time interaction was found for craving, *F*(6, 366) = 8.61, *P* < .001, η^2^*g* = .023, indicating differing trajectories over time (Figure 2, left). However, no significant group differences were found at individual timepoints (*p*-adjusted > .101). There was a significant main effect of time, *F*(3, 366) = 40.30, *P* < .001, η^2^*g* = .053, but no main effect of group, *F*(2, 122) = 1.11, *P* = .33, η^2^*g* = .015.

The analysis was rerun with age as a covariate in order to ­investigate whether it would change the outcomes of the analyses. This analysis yielded no significant main effects of age (see Supplementary Material 4).

### Expectations

The effect of the group on the expectations regarding change in hunger after the sham vagus nerve stimulation was significant, χ2(2) = 39.22, *P* < .001, generalized eta squared (η^2^*g*) = .30. Post-hoc comparisons with Bonferroni corrections revealed that the hunger-decreasing group, *M* = –11.7, *SD *= 15.0, expected to experience significantly less hunger, compared to the hunger-increasing, *M *= 14.0, *SD *= 18.8; *z* = –5.99, *p*-adjusted < .001, and control groups, *M *= 7.63, *SD *= 14.7; *z *= –4.49, *p*-adjusted < .001. The difference between hunger-increasing and control groups was not significant, *z *= 1.63, *p*-adjusted = .153.

**Figure 3:**
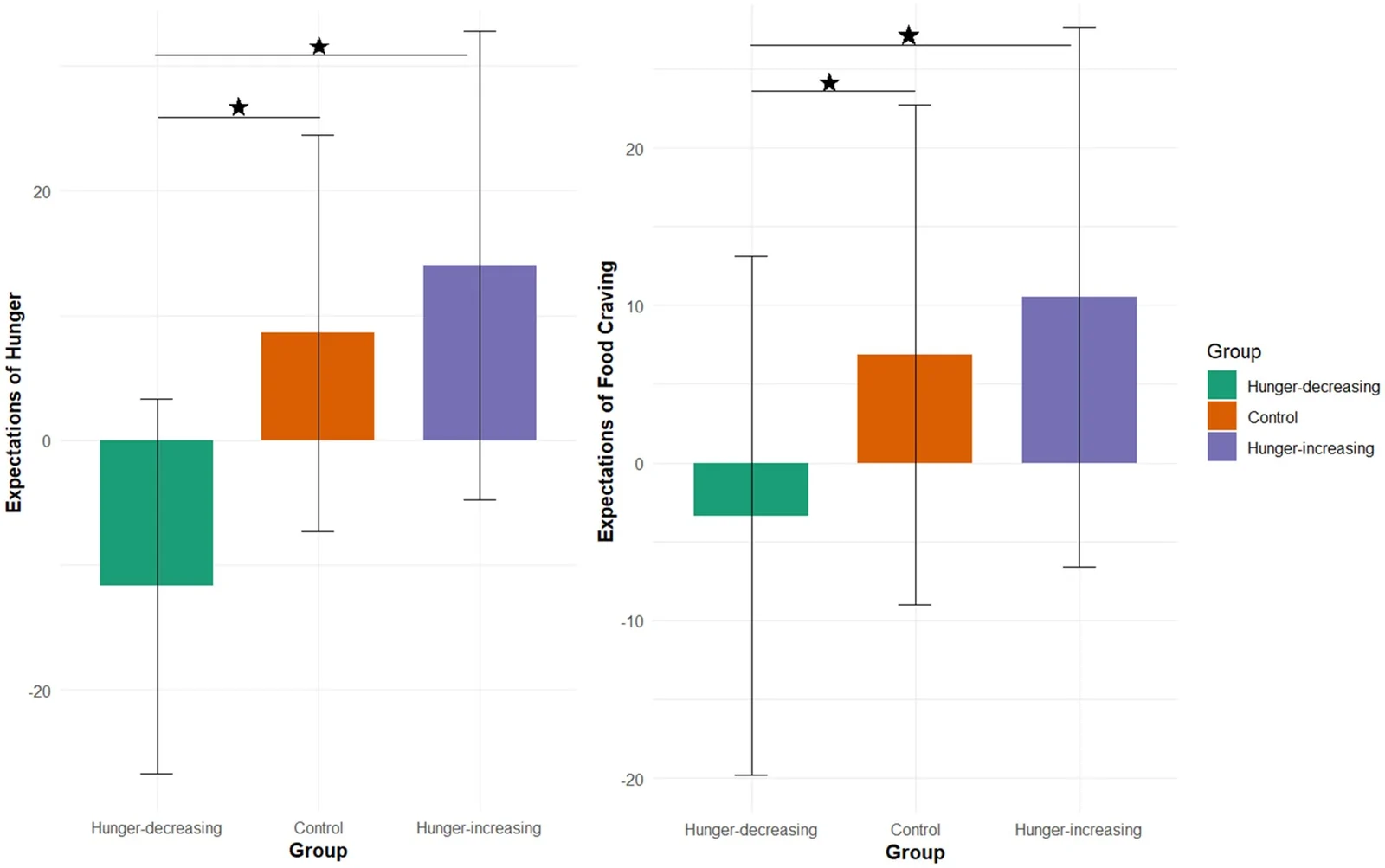
The means and SEs of the expectations per group.

The effect of the group on the expectations regarding change in food craving after the sham vagus nerve stimulation was significant, *F*(2, 122) = 7.96, *P* < .001, η^2^*g *= 0.058. Hunger-decreasing group expected to experience significantly less food craving, ­compared to the hunger-increasing, mean difference = –13.87, 95% CI [–22.52, –5.22], *p*-adjusted < .001, and control groups, mean difference = 10.18, 95% CI [1.80, 18.56], *p*-adjusted = .012. The difference between hunger-increasing and control groups was not significant, mean difference = –3.69, 95% CI [–12.39, 5.01], *p*-adjusted = .574. The means and SEs of the expectations per group are presented in Figure 3.

### Expectations as a mediator

Comparing the hunger-decreasing and control groups, participants in the hunger-decreasing group expected less hunger (*b *= 19.31, *P* < .001). However, expectations did not significantly predict hunger change scores (*b* = –0.16, *P* = .089). The direct effect of the hunger-decreasing group on hunger change was not significant (*b* = –3.22, *P* = .412, 95% CI [–10.96, 4.52]), nor was the indirect effect through expectations (*b* = –3.02, 95% CI [–6.83, 0.16]).

In the hunger-increasing group versus control, participants did not significantly differ from the control group in their expectations of hunger (*b* = –6.40, *P* = .078). Expectations were not significantly associated with hunger change scores (*b* = –0.16, *P* = .089). The hunger-increasing group showed a significant direct effect on hunger change (*b *= 9.28, *P* = .013, 95% CI [2.01, 16.55]), but the indirect effect through expectations was not significant (*b *= 1.00, 95% CI [−0.24, 3.17]).

For craving, the hunger-decreasing group expected greater craving reduction than controls (*b *= 10.18, *P* = .005), but expectations did not significantly predict craving change (*b* = –0.07, *P* = .403). The direct effect of the hunger-decreasing group on ­craving change was significant (*b* = –12.54, *P* < .001, 95% CI [–19.13, –5.95]), whereas the indirect effect through expectations was not significant (*b* = –0.71, 95% CI [–2.75, 0.79]).

In the hunger-increasing group, participants did not significantly differ from the control group in craving expectations (*b* = –3.69, *P* = .316). Expectations were not significantly associated with craving change (*b* = –0.07, *P* = .403). The direct effect of the hunger-increasing group on craving change was not significant (*b *= 5.62, *P* = .097, 95% CI [–1.03, 12.27]), and the indirect effect through expectations was not significant (*b *= 0.26, 95% CI [−0.54, 1.41]).

### Food desirability and food choice

Table 3 depicts the average ratings for Food Desirability and Food Choice tasks of the 3 groups. The analysis revealed a non-significant effect of the group on the average ratings of the desirability of healthy foods, *F*(2, 120) = 0.402, *P* = .67, η^2^ = 0.01. This was also the case for the unhealthy food items, *F*(2, 120) = 2.30, *P* = .105, η^2^ = 0.01. There was also no difference between the groups in the proportion of healthy food items chosen, *F*(2, 120) = 0.83, *P* = .44, η^2^ = 0.01.

**Table 3:** Means and standard deviations of task variables.

Variable	Overall		Hunger-decreasing		Hunger-increasing		Control	
	M	(SD)	M	(SD)	M	(SD)	M	(SD)
**Healthy Food desirability rating**	43.7	(16.6)	43.5	(17.4)	45.6	(16.9)	42.3	(15.7)
**Unhealthy Food desirability rating**	43.8	(18.7)	40.3	(18.0)	48.9	(19.4)	42.7	(18.0)
**Proportion of chosen healthy foods**	0.45	(0.25)	0.49	(0.24)	0.42	(0.26)	0.43	(0.26)

### Exploratory analyses

As shown in Electronic Supplementary Material 3  Table 1, hunger and craving scores correlated positively with unhealthy food ratings (ρ  =  0.28, *P* < .001; ρ  =  0.23, *P* = .010), but not with healthy food ratings. Both hunger and craving were negatively correlated with the proportion of healthy foods chosen (ρ  =  0.18, *P* = .049; ρ  =  0.26, *P* = .004). Hunger and craving also showed moderate positive correlations at each timepoint, as well as the mean score (ρ’s > .51, *P* < .001) (Supplementary Material 3 Table 2).

## Discussion

The goal of the present study was to investigate whether it is possible to affect hunger and food craving by verbal suggestions. Particularly, the study investigated whether hunger-decreasing and hunger-increasing suggestions about a sham vagus nerve stimulation affected hunger, food craving, expectations, food desirability, and food choice in healthy, not obese participants. Findings indicated that hunger-decreasing placebo suggestions significantly reduced overall food craving, while hunger-increasing suggestions led to significantly more overall hunger in the participants. Analysis of the timeline of the experiment revealed that hunger-decreasing and hunger-increasing groups differed in hunger at the end of the experiment after the hunger-inducing food imagery and food viewing tasks. Moreover, no effect of the verbal suggestions on food desirability and food choice was found.

The total change in hunger was significantly higher in the hunger-increasing group compared to the control and hunger-decreasing groups, while the hunger-decreasing group did not differ from the control one. Therefore, it seems that hunger-increasing placebo suggestions had a more robust effect on hunger than hunger-decreasing placebo suggestions. This finding contradicts the results of a similar experimental study by Hoffmann [8] which, in opposition, found no significant hunger-increasing effect of a placebo pill, but found a significant hunger-decreasing effect. The study of Khalid et al. [36], on the other hand, found an increase in hunger in both hunger-increasing and hunger-decreasing suggestions groups. The reasons for these inconsistencies remain unclear. One potentially relevant factor is the difference in placebo modalities used across studies (eg, placebo pills, water, or vagus nerve stimulation). Prior research indicates that placebo effects can vary depending on the mode of administration [37]. It is therefore possible that vagus nerve stimulation, which produces unfamiliar and ambiguous bodily sensations, may be more prone to nocebo-like responses. Such sensations can heighten bodily vigilance and increase the likelihood of negatively interpreting internal cues. In addition, ­electrical stimulation may carry medical or invasive connotations, potentially inducing more cautious or negative expectations. These combined factors may bias participants toward perceiving increased hunger, whereas placebo pills—familiar and culturally associated with therapeutic benefit—may lead to more positive or hunger-decreasing expectations. Further research directly comparing placebo modalities is needed to test this interpretation.

At the same time, the pattern of the findings regarding craving is different; the total craving score was lower in the hunger-decreasing than in the control group, while the hunger-increasing group did not differ from the control group in the total craving score. Therefore, hunger-decreasing suggestions were successful in reducing food craving, while hunger-increasing suggestions did not enhance craving. It remains unclear why there is this discrepancy with the hunger data. The 2 concepts are highly related, however, craving measured in the current study was more specific than hunger: craving (desire to eat) was assessed specifically for participants’ favorite meal. Future research should look more into the relationship between these 2 constructs and investigate which of them should actually be targeted for successful weight management.

Looking at the change in time, the found effects seem to be visible when measured after the three tasks: food imagery task, food rating task and food choice tasks. Imagining favorite food as well as looking at pictures of food have been previously demonstrated to have large hunger-boosting effects [15, 24, 38]. These hunger- and craving-increasing effects are also visible in the data as a significant effect of time: both hunger and craving increased after the imagery task (however, it is worth mentioning that participants could have also experienced more hunger during the session as more time passed since their last meal). Those results are in line with a previous study that demonstrated that open-label placebo prevented hunger increase in response to food image exposure [9]. It could be that expectations induced by suggestions play a particularly large role in situations when hunger is externally triggered. If it is the case, placebo interventions might have potential for individuals struggling with excessive hunger triggered by external stimuli, an increasingly common issue in today’s obesogenic environment, characterized by the widespread availability of high‑calorie foods and environmental cues such as pervasive food marketing, prominent visual displays of palatable foods, and potent sensory triggers.

Furthermore, the manipulation check demonstrated that the suggestions given regarding the sham vagus nerve stimulation were partially effective in changing the expectations of the participants. Participants in the hunger-decreasing group expected to experience less hunger and craving, whereas participants in the hunger-increasing group did not expect greater hunger and craving than participants in the control group. Possibly, suggestions implying improvement or symptom reduction are generally more aligned with participants’ intuitive expectations about medical interventions, which could have led to a stronger shift in expectations in the hunger-decreasing group. Moreover, we did not confirm our hypothesis that expectations would mediate the effects of suggestions on hunger. This contradicts the theory of placebo effects that describes expectations as the main mechanism [39, 40]. However, this is not the only study that does not find such a mediation effect [41, 42]. Verbal suggestions may have influenced hunger through processes that were not fully captured by the single explicit expectation item. Placebo effects can arise from implicit learning, affective shifts, or attentional changes [39, 43], which operate alongside or even independently of consciously reported expectations.

However, it is important to note that the effects found in this study are quite small, around 10 points on a 100 Likert scale. This is comparable to the effects found in the previous research on placebo effects on hunger [8, 36]. However, literature shows that longer interventions tend to lead to higher placebo effects [44], therefore, it is possible that a single, short, and sham vagus nerve stimulation was not enough to elicit large effects. Research also demonstrates that people who might benefit the most from interventions, such as patient groups, tend to experience larger placebo effects [1]. In this study, healthy participants were included, with no obesity and other metabolic disorders. It is possible that they were not motivated to modify their hunger, which could have led to the smaller effects of the suggestions.

Furthermore, no effect of suggestions was found on how desirable participants found various food items and on their food choice, even though both of these measures were correlated with hunger. Only one previous study looked at the potential effects of placebo suggestions on food choice [36] and found that participants who received hunger-increasing suggestions tended to choose more food items as desirable than participants in the control group. However, the task used in the current study was different, and instead of accepting or rejecting various food items as in the study of Khalid et al. [36], participants had to make a choice between healthy and unhealthy items. Therefore, the current study looked at the effect of the healthiness of chosen foods rather than the number of chosen foods. Possibly, the effects of suggestions found in this study were not large enough to affect the food choice and desirability.

Several limitations of this study should be noted. Participants were healthy young volunteers with a mean BMI of 22, lower than the Dutch population average of 25.5 [45], limiting generalizability. The lower weight in this sample, could also suggest less interest in the sham treatment for reducing hunger, which may have affected the strength of the hunger-decreasing and hunger-increasing effects. Moreover, participants were university students and only information on their age and gender was collected, which limits generalizability to other population groups. Furthermore, the timing of the experimental sessions was not standardized between participants for practical reasons, which could have influenced the results due to appetite and metabolic responses. However, participants refrained from eating before the study and no baseline differences were found in hunger and food craving between the groups, suggesting minimal effects on the results. The study was single-blinded, which could lead to unintentional experimenter influence. To minimize the risk, experimenters followed a strict protocol and all questionnaires were administered to participants on a computer, ensuring no influence in their responses from the experimenters. Additionally, the study was performed in laboratory settings, allowing precise manipulations, but may limit translatability to real-life settings. It remains unclear how the placebo manipulations would have affected hunger long-term and food choices outside the lab. A further limitation concerns the measurement of hunger and food craving. Although these constructs are theoretically and empirically distinct, in the present study they were each assessed using single‑item measures with similar phrasing, which may have led participants to respond in ways that did not fully differentiate between general hunger and craving for a specific food. This possibility is reflected in the moderate-to-strong correlations observed between the two items across time points, suggesting partial overlap in how participants interpreted the questions. Future research using validated multi‑item scales or more discriminative measurement approaches will be needed to more precisely capture the distinction between hunger and food craving. Finally, in the Food Imagery Task, we used food photographs sourced from Google Images. This approach was chosen for practical reasons, as images needed to be selected rapidly during the experimental session. However, because these images were not standardized, their variability may have influenced the consistency or strength of the craving and hunger responses they elicited. Nevertheless, we observed significant increases in both hunger and craving following the task, suggesting that the imagery was effective despite its lack of standardization.

These findings provide preliminary support that expectations, shaped by verbal suggestions, can modulate subjective hunger and food craving—even without an active intervention. This highlights the potential relevance of placebo mechanisms in appetite regulation. Although the study involved healthy volunteers, future research should investigate whether such effects are amplified in populations more motivated to manage appetite, such as individuals with overweight or obesity. Prior research suggests that clinical populations often experience stronger placebo responses, particularly when interventions align with personal goals or health concerns [1]. If similar effects emerge, harnessing expectation-based strategies could complement weight management interventions. For instance, increasing positive expectations through therapeutic framing, psychoeducation, or even open-label placebos may improve behavioral or dietary outcomes. Additionally, targeting moments when hunger is externally triggered (eg, by food cues) could be particularly effective for such interventions. Integrating expectation-based approaches into clinical practice may offer a low-cost, low-risk addition to current appetite and weight interventions.

This study demonstrated that verbal suggestions about a sham hunger-modulating treatment can influence subjective experiences of hunger and food craving in healthy individuals. Specifically, hunger-decreasing suggestions reduced craving and momentary hunger after exposure to food cues, while hunger-increasing suggestions increased overall hunger. These findings underscore the potential of expectation-driven interventions to influence appetite-related experiences, particularly in contexts where hunger is externally triggered. Although the effects observed were modest and did not translate to changes in food desirability or choice, these results contribute to the growing body of evidence suggesting that cognitive factors, such as expectations, can shape eating-related experiences. Future studies should explore whether these effects generalize to clinical populations, such as individuals with overweight and obesity, and whether they can be harnessed in real-world interventions aimed at regulating appetite and supporting healthier eating behavior.

## Supplementary Material

kaag036_Supplementary_Data

## OSF preregistration

The study was pre-registered on the Open Science Framework platform and can be found via this link: https://osf.io/uj6f7.
