# Supplementary material for: Expectation shapes hunger and craving: placebo effects of verbal suggestion on food-related experiences
Source: Ann Behav Med. 2026 Jun 19;60(1):kaag036. doi: 10.1093/abm/kaag036 (PMC13282074; doi:10.1093/abm/kaag036)
Supplement: kaag036_Supplementary_Data [file kaag036_supplementary_data.zip › Revised_Electronic Supplementary Material 3.docx]

**Electronic Supplementary Material 3**

**Table 1.** Correlations between hunger, craving, food desirability, and food choice task variables

| Variable pairs | *rho* | *p* |
| --- | --- | --- |
| Hunger vs Healthy food desirability rating | 0.11 | .242 |
| Hunger vs Unhealthy food desirability rating | 0.28 | .001* |
| Craving vs Healthy food desirability rating | –0.02 | .859 |
| Craving vs Unhealthy food desirability rating | 0.23 | .010* |
| Hunger vs Proportion healthy foods chosen | –0.18 | .049* |
| Craving vs Proportion healthy foods chosen | –0.26 | .004* |

Note: Asterisk (*) indicates *p* < .05

**Table 2.** Hunger and Craving scores correlation

| Hunger vs Craving | *rho* | *p* |
| --- | --- | --- |
| Baseline | 0.51 | < .001 |
| T1 | 0.65 | < .001 |
| T2 | 0.72 | < .001 |
| T3 | 0.66 | < .001 |
| Mean score | 0.63 | < .001 |
