# Supplementary material for: Expectation shapes hunger and craving: placebo effects of verbal suggestion on food-related experiences
Source: Ann Behav Med. 2026 Jun 19;60(1):kaag036. doi: 10.1093/abm/kaag036 (PMC13282074; doi:10.1093/abm/kaag036)
Supplement: kaag036_Supplementary_Data [file kaag036_supplementary_data.zip › Finalized Electronic Supplementary Material 4.docx]

**Electronic Supplementary Material 4**

For the exploratory analyses, the change scores of hunger or food craving were used as the outcome variable. A hierarchical regression approach was used, with baseline craving and group entered in the first model, individual difference variables (e.g., affect, BMI, body image, personality traits, happiness) added in the second model, and the interactions between group and these individual differences added in the third model.

The regression model with baseline characteristics as predictors of hunger, explained a low proportion of variance (*R²*-adjusted = 0.20, *p* = .007), with baseline hunger as the only significant predictor (*p* < .001). No significant effects were found for personality traits, affect, BMI, happiness, or body image. Only one significant group interaction was found, namely group x negative affect (*p* = .035). Simple slopes analysis showed that in the hunger-decreasing group, a higher negative affect at baseline predicted a larger reduction in hunger (*b* = -2.62, *SE* = 1.18, *t* = -2.22, *p* = 0.03), while the other groups showed no significant association (*ps* >.41).

For food cravings, the model explained a moderate amount of variance (*R²*-adjusted = 0.30, *p* < .001), again with baseline craving as the strongest predictor (*p* < .001). No other variables or interactions were significant, except for a group × body image interaction (*p* = .015). Simple slopes analysis showed that in the hunger-decreasing group, more negative body image was associated with greater cravings (*b* = 0.57, *SE* = 0.16, *t* = 3.35, *p* < .001), while no such association was found in the other groups (*p*s > .73).

Additionally, the main ANOVA analyses for hunger and food craving were rerun with age as a covariate in order to investigate the potential effect of age in group differences. For the hunger ANOVA analysis, the main effect of group remained insignificant, *F*(2, 105) = 1.00, *p* = .372, η²*g* = 0.012, and the main effect of time was significant, *F*(3, 315) = 26.41, *p* < .001, η²*g* = 0.041. The group × timepoint interaction also remained significant, *F*(6, 315) = 5.01, *p* < .001, η²*g* = 0.010. The main effect of age (*p* = .438), as well as the age × timepoint interaction (*p* = .080) were both insignificant.

Similarly, the food craving ANOVA analysis did not significantly change with age added as a covariate. The main effect of group was insignificant, *F*(2, 105) = 0.11, *p* = .90, η²*g* = 0.001, and the main effect of time was significant, *F*(3, 315) = 15.88, *p* < .001, η²*g* = 0.021. The group × timepoint interaction was significant, *F*(6, 315) = 8.17, *p* < .001, η²*g* = 0.020. The main effect of age (*p* = .079), as well as the age × timepoint interaction (*p* = .070) were insignificant.
