# Supplementary material for: Expectation shapes hunger and craving: placebo effects of verbal suggestion on food-related experiences
Source: Ann Behav Med. 2026 Jun 19;60(1):kaag036. doi: 10.1093/abm/kaag036 (PMC13282074; doi:10.1093/abm/kaag036)

**Electronic Supplementary Material 1**

**Figure 1.** Study procedure.

**Alt text.** Study procedure timeline; baseline questionnaire is followed by group allocation (hunger-increasing, hunger-decreasing, control) and vagus nerve stimulation. Questionnaires were administered before and after the Food Imagery Task, and once more after the Food Desirability Task and Food Choice Task.


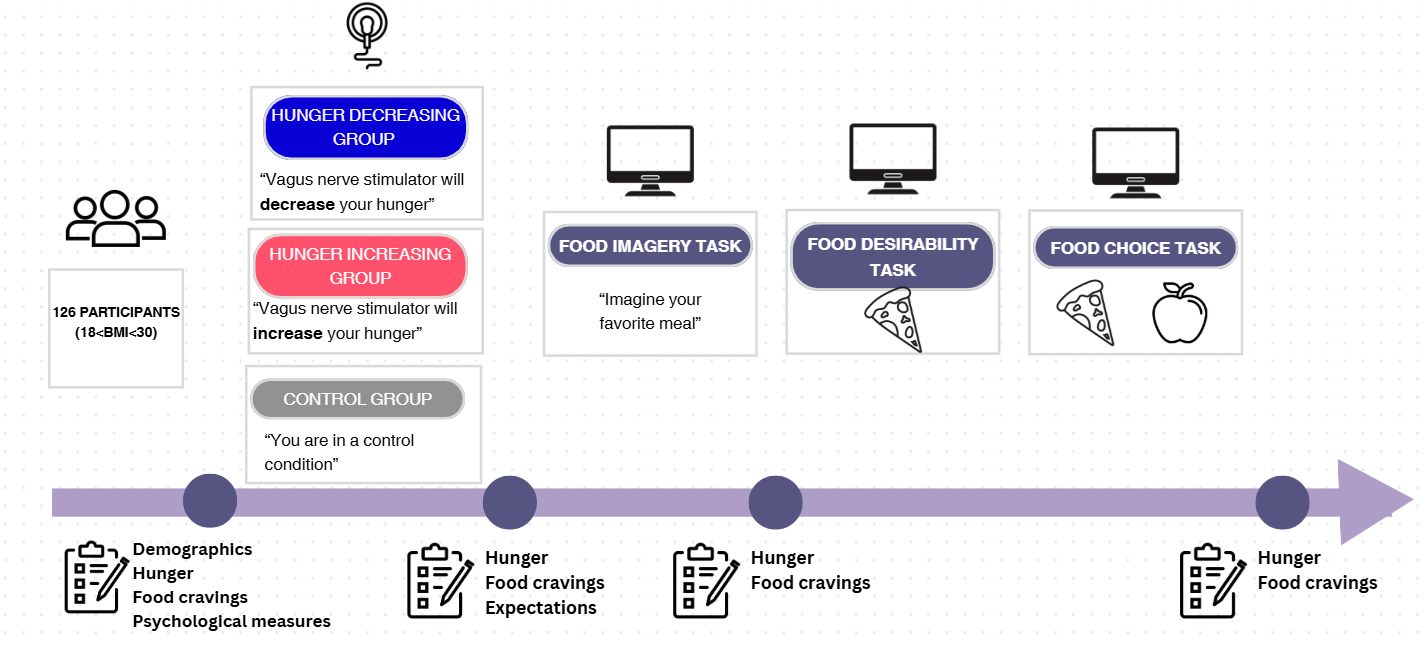

Supplement: kaag036_Supplementary_Data [file kaag036_supplementary_data.zip › Revised_Electronic Supplementary Material 1.docx]
