# Supplementary material for: Expectation shapes hunger and craving: placebo effects of verbal suggestion on food-related experiences
Source: Ann Behav Med. 2026 Jun 19;60(1):kaag036. doi: 10.1093/abm/kaag036 (PMC13282074; doi:10.1093/abm/kaag036)
Supplement: kaag036_Supplementary_Data [file kaag036_supplementary_data.zip › Revised_Electronic Supplementary Material 2.docx]

**Electronic Supplementary Material 2**

The script of suggestions in the hunger-decreasing placebo group.

“After that I will attach a vagus nerve stimulator to your ear. It is this small device [show the stimulator to the participant] that sends light electrical pulses. These pulses go through the skin and stimulate the vagus nerve that goes behind your ear, as you can see on this picture [show the picture with the ear]. Vagus nerve connects your stomach with your brain and its activity makes you feel less hungry. Stimulation of the vagus nerve can have positive effects on your brain as it influences the production of certain neurotransmitters. Research has shown that using the vagus nerve stimulation not only has beneficial effects on anxiety, but also has an effect on our hunger. Previous research has demonstrated that hunger decreases strongly after stimulation of the vagus nerve. Most participants in other studies reported that it has decreased their hunger. Therefore, we expect that you will experience less hunger after the stimulation”.

The script of suggestions in the hunger-increasing placebo group.

“After that I will attach a vagus nerve stimulator to your ear. It is this small device [show the stimulator to the participant] that sends light electrical pulses. These pulses go through the skin and stimulate the vagus nerve that goes behind your ear, as you can see on this picture [show the picture with the ear]. Vagus nerve connects your stomach with your brain and its activity makes you feel more hungry. Stimulation of the vagus nerve can have positive effects on your brain as it influences the production of neurotransmitters and decreases anxiety. However, as a side effect the stimulation seems to increase hunger. Previous research has demonstrated that hunger increases strongly after stimulation of the vagus nerve. Most participants in other studies reported that it has increased their hunger. Therefore, we expect that you will experience more hunger after the stimulation”.

The script of suggestions in the control group.

“You have been assigned to the control group, so it means that you will receive no real vagus nerve stimulation. However, to be consistent with other groups, I will still ask you to hold the stimulator against your skin. It is this small device [show the stimulator to the participant] that sends light electrical pulses. But you will be holding it at the point where there is no nerve”.
